# Supplementary material for: Familial Combined Hyperlipidemia (FCH) Patients with High Triglyceride Levels Present with Worse Lipoprotein Function Than FCH Patients with Isolated Hypercholesterolemia
Source: Biomedicines. 2020 Jan 6;8(1):6. doi: 10.3390/biomedicines8010006 (PMC7168323; doi:10.3390/biomedicines8010006)
Supplement: Supplementary file 1 [file biomedicines-08-00006-s001.pdf]

Supplemental data

Supplemental Table S1. Treatments for every patient.

NTG group		HTG group	
Drug	Dose (mg/day)	Drug	Dose (mg/day)
Atorvastatin	40	Atorvastatin	40
Pravastatin	20	Atorvastatin	20
Atorvastatin	20	Atorvastatin	40
Atorvastatin	40	Atorvastatin	40
Atorvastatin	20	Rosuvastatin	5
Atorvastatin	40	Atorvastatin	40
Atorvastatin	80	Atorvastatin	40
Atorvastatin	20	Atorvastatin	20
Atorvastatin	40	Atorvastatin	40
Atorvastatin	20	Simvastatin	40
Simvastatin	40	Atorvastatin	20
Atorvastatin	20	Simvastatin	40
Atorvastatin	40		

Supplemental Table S2. Correlations between plasma triglyceride levels and the rest of analyzed parameters. Analysis was performed with the Spearman correlation test using the whole population (n=40). Significant correlations are marked in bold type.

Plasma parameters	r	P
**Total cholesterol**	0.6130	<0.0001
**VLDL-c**	0.9866	<0.0001
**LDL-c**	0.4845	0.0024
**HDL-c**	- 0.7099	<0.0001
**apoB**	0.7759	<0.0001
**CRP**	0.4435	0.0067
Leptin	0.0242	0.8851
Adiponectin	0.0675	0.6873
BMI	0.2212	0.2016
Age	0.1178	0.4811
Lp-PLA2 distribution		
**Total Lp-PLA2 (activity)**	0.4237	0.0080
Lp-PLA2 in HDL (activity)	0.2152	0.2009
% Lp-PLA2 in HDL	- 0.2566	0.1308
**% Lp-PLA2 in apoB**	0.3948	0.0156
apoJ distribution		
% apoJ not bound to lipoproteins	0.2932	0.0782
% apoJ bound to lipoproteins	- 0.2932	0.0782
**% apoJ VLDL**	- 0.3202	0.0500
**% apoJ LDL**	- 0.3430	0.0350
% apoJ HDL	- 0.979	0.5586
apoJ	0.1852	0.2795
VLDL composition		
Total cholesterol	-0.2985	0.0687
Triglyceride	0.0381	0.8475
Phospholipids	0.1020	0.5365
Esterified cholesterol	-0.3156	0.0503
Free cholesterol	-0.0062	0.9702
Protein	-0.1560	0.3495
**apoB**	-0.3308	0.0425
apoE	-0.2665	0.1011
**apoC-III**	0.7516	0.0001
LDL composition		
**Total cholesterol**	-0.6714	0.0001
**Triglyceride**	0.4139	0.0088
**Phospholipids**	-0.5225	0.0006
**Esterified cholesterol**	-0.3807	0.0168
**Free cholesterol**	-0.6440	0.0001
**Protein**	0.5905	0.0001
**apoB**	0.5874	0.0001
apoE	-0.2337	0.1522
**apoC-III**	0.4349	0.0064
Qualitative properties of LDL		
**LDL size**	- 0.6146	<0.0001
**LDL(-)**	0.3303	0. 0459
**Lag time**	- 0.3684	0.0294
HDL composition		
**Total cholesterol**	-0.4651	0.0029
**Triglyceride**	0.6191	0.0001
**Phospholipids**	-0.3764	0.0182
**Esterified cholesterol**	-0.4210	0.0076
Free cholesterol	-0.2763	0.0887
Protein	0.2080	0.2038
apoA-I	-0.3099	0.0583
apoA-II	0.2356	0.1487
**A-I/A-II**	-0.3420	0.0331
**apoC-III**	0.6931	0.0001
apoE	-0.1667	0.3170
Qualitative properties of HDL		
**% HDL2**	- 0.6244	<0.0001
Efflux	- 0.1284	0.3204
Increase of lag time	0.2380	0.1686
Inhibition IL6 release	0.1640	0.4897
